# Effects of Coffee on Gut Microbiota and Bowel Functions in Health and Diseases: A Literature Review

**DOI:** 10.3390/nu16183155

**Published:** 2024-09-18

**Authors:** Sena Saygili, Shrilakshmi Hegde, Xuan-Zheng Shi

**Affiliations:** 1 John Sealy School of Medicine, University of Texas Medical Branch, Galveston, TX 77555, USA; sesaygil@utmb.edu; 2 Department of Tropical Disease Biology, Liverpool School of Tropical Medicine, Liverpool L3 5QA, UK; 3 Department of Internal Medicine, University of Texas Medical Branch, Galveston, TX 77555, USA

**Keywords:** coffee, caffeine, microbiota, dysbiosis, gastrointestinal tract, motility, secretion, gut–brain axis, absorption, immunity

## Abstract

**Background and objectives:** As one of the most popular beverages in the world, coffee has long been known to affect bowel functions such as motility, secretion, and absorption. Recent evidence obtained in human and animal studies suggests that coffee has modulating impacts on gut microbiota. We aim to present an overview of the specific effects of coffee on gut microbiota composition, diversity, and growth. We will also critically review the impacts of coffee on bowel functions in health and diseases and discuss whether gut microbiota play a role in the coffee-associated functional changes in the gastrointestinal tract. **Methods:** We searched the literature up to June 2024 through PubMed, Web of Science, and other sources using search terms such as coffee, caffeine, microbiota, gastrointestinal infection, motility, secretion, gut–brain axis, absorption, and medication interaction. Clinical research in patients and preclinical studies in rodent animals were included. **Results:** A majority of the studies found that moderate consumption of coffee (<4 cups a day) increased the relative abundance of beneficial bacterial phyla such as Firmicutes and Actinobacteria and decreased Bacteroidetes. Moderate coffee consumption also increased *Bifidobacterium* spp. and decreased the abundance of Enterobacteria. Coffee consumption is reported to increase gut microbiota diversity. Although the effects of coffee on bowel functions have been known for a long time, it is not until recently that we have recognized that some of the effects of coffee may be partly due to its impacts on microbiota. **Conclusions:** The current literature suggests that moderate coffee consumption has beneficial effects on oral and gut microbiota and motility function. However, excessive coffee intake (>5 cups a day) is implicated in reflux disorders, periodontal diseases, and progression of Crohn’s disease. Further research in the field is needed, as there are many conflicting results regarding the impacts of coffee in the gastrointestinal tract.

## 1. Introduction

Coffee is one of the most popular beverages globally, not only for its taste and aroma but also for its beneficial effects in multiple biological processes in health and diseases [1]. Historically, coffee consumption has been thought to have largely negative health outcomes, but growing evidence now suggests that coffee has important beneficial impacts on several aspects of health [2]. The physiological impacts of coffee are attributed to its complex composition, which consists of more than 1000 different bioactive compounds such as caffeine, kahweol, chlorogenic acid, cafestol, and different micronutrients like magnesium, potassium, and vitamin E [3,4]. Recent studies have implicated coffee as a immunomodulator, antiobesity, antidiabetic, hepatoprotective, antioxidant, antigenotoxic, and cell proliferation inhibitor [2,5,6]. Despite its prohealth benefits, excessive coffee drinking has been associated with some cardiovascular and gastrointestinal adverse effects and dysfunctions [7,8]. Hence, it is suggested to consume only a moderate amount of coffee (3–4 cups per day) and limit the caffeine intake to equal or less than 200 mg/day to utilize the health benefits while limiting any possible adverse health effects [9,10].

One of the most direct effects of coffee intake is on the gut microbiota. Microbiota is considered second genome of the human body and affects a wide array of body functions and wellbeing [11,12]. With an estimated size of 10 [13] microbial cells in the gastrointestinal (GI) tract, the gut microbiota is the largest reservoir of microbes within the body, though there are also oral, respiratory, skin, and reproductive tract microbiotas that have proven significant physiological role in the different organs [13,14]. Although the gut microbiota contains fungi, viruses, and bacteriophages, it consists predominantly of commensal and symbiotic bacteria. Firmicutes, Bacteroidetes, Proteobacteria, and Actinobacteria are the four major phyla of bacteria in the gut, with Firmicutes and Bacteroidetes accounting for nearly 90% of the relative abundance of the gut microbiota. The microbiota has been known to influence a wide range of physiological processes in the gut such as metabolism, digestion, absorption, motility, secretion, permeability, enteric nervous system (ENS), gut associated lymphatic tissue (GALT), immunity, infections, and inflammatory responses [13,15,16]. In addition, it also has long reaching effects on the systemic processes like cardiovascular, neurological, and musculoskeletal systems and even in the development of the immune system [15,16,17]. Growing evidence obtained in human and animal studies suggests that coffee has modulating impacts on gut microbiota and gastrointestinal functions such as motility, secretion, absorption, and immune response. Some of the coffee effects on gastrointestinal functions may partly be through its impacts on microbiota. In this article, we will look closely into the specific effects of coffee on gut microbiota composition, diversity, and growth. We will also review the effects of coffee on selected gastrointestinal functions in health and diseases and discuss whether the gut microbiota has a role in these coffee-associated functional changes in the gastrointestinal tract. We searched the literature up to June 2024 through PubMed, Web of Science, and other sources using search terms such as coffee, caffeine, microbiota, gastrointestinal infection, motility, secretion, gut–brain axis, absorption, and medication interaction. Clinical research in patients and preclinical studies in rodent animals were included.

## 2. Effects of Coffee on the Gut Microbiota

### 2.1. Effect of Coffee on Microbiota Composition

The gut microbiota is in close harmony with its microenvironment. Any change in diet, lifestyle, and health conditions can bring out significant changes in microbiota structure and composition, resulting in dysbiosis. Due to the intricated role of gut microbiota in several physiological processes, dysbiosis leads to clinical implications [18]. Several studies in animals and using human samples investigated the gut microbiota changes in response to coffee intake. Table 1 summarizes the specific changes of the gut microbiota in response to coffee consumption or treatments with coffee components in humans and animals. A majority of the studies investigating human fecal samples reported that coffee consumption increased the relative abundance of Firmicutes and Actinobacteria [19,20,21,22,23,24]. On the contrary, Ref. [25] reported a decrease in Firmicutes. Coffee consumption was reported to decrease relative abundance of *Bacteroidetes* in the gut [21] (Table 1), whereas another study reported that soluble coffee fiber increased the relative abundance of *Bacteroides*–*Prevotella*, a genus belonging to the Bacteroidetes phylum, by 60% [26]. A human volunteer study revealed that consumption of 3 cups of coffee for 3 weeks can mediate significant changes in the microbiota composition and increase the abundance of beneficial Actinobacteria and *Bifidobacterium* spp. in the gut [27].

Animal studies have the advantages to not only keep strict control and study groups, but also to investigate cellular and molecular mechanisms of coffee effects. A study in a rodent model found that long-term coffee consumption led to an increase in short chain fatty acids (SCFAs) producing bacteria such as *Faecalibacterium* and *Bifidobacterium longum* and a parallel decrease in *Prevotella*, resulting in a subsequent increase in SCFA levels [28] (Table 1). Coffee intake can mediate changes in microbiota through caffeine and/or other coffee components or metabolites like micronutrients and phenolic compounds. For instance, caffeine consumption partially reversed amoxicillin-induced dysbiosis in the murine gut compared to decaffeinated coffee, suggesting a role of caffeine in the effect [29]. However, separate studies have reported no significant difference in the effect of caffeinated and decaffeinated coffee on gut microbiota, suggesting that other components in coffee are also important in mediating coffee effects on gut microbiota [30,31].

**Table 1:** Effect of coffee on microbiota composition in human and animal studies.

Samples Studied	Treatments	Increased Phyla/Class/Genera	Decreased Phyla/Class/Genera	References
Human fecal samples	Mannooligosaccharides from coffee	↑ Actinobacteria	↓ Firmicutes; ↓ Lactobacillus	Umemura et al., 2004 [25]
Human fecal samples	Coffee fibres	↑ *Bacteroides*; ↑ *Prevotella grp*		Gniechwitz et al., 2007 [26]
Human fecal samples	Three cups of coffee daily for 3 days	↑ Actinobacteria; ↑ *Bifidobacterium* spp.		Jaquet et al., 2009 [27]
Human fecal samples	Coffee extract	↑ Actinobacteria; ↑ Firmicutes; ↑ *Bifidobacterium* spp.	↓ Proteobacteria; ↓ *E. coli*	Benitez et al., 2019 [22]
Human fecal samples	Coffee extract and chlorogenic acids	↑ Actinobacteria; ↑ Firmicutes; ↑ *Bifidobacterium* spp.		Tomas-Barberan et al., 2014 [20]
Human fecal samples	Chlorogenic acid (C-QA)	↑ Actinobacteria; ↑ Firmicutes; ↑ *Bifidobacterium* spp.	↓ Bacteroidetes	de Cosío-Barr´on et al., 2020 [21]
Human fecal samples	Nescafe coffee extracts	↑ Actinobacteria; ↑ Firmicutes; ↑ *Bifidobacterium* spp.		Mills et al., 2015 [19]
Human fecal samples	Spent coffee	↑ Bacteroidetes; ↑ Firmicutes; ↑ *Barnesiella*; ↑ *Butyricicoccus*; ↑ *Veillonella*	↓ Actinobacteria; ↓ *Faecalibacterium*; ↓ *Ruminococcus*; ↓ *Blautia*	Perez-Burillo et al., 2020 [23]
Mice fecal samples	Coffee and amoxicillin	↑ Proteobacteria; ↑ *Burkholderiaceae*	↓ *Burkholderia cepacia*	Diamond et al., 2021 [29]
Rat fecal samples	Caffeinated and decaffeinated coffee		↓ *Enterobacteria*; ↓ gamma-Proteobacteria	Hegde et al., 2022 [31]
Rat fecal samples (paradoxical sleep deprivation)	Caffeinated and decaffeinated coffee	↑ *Akkermansia*; ↑ *Klebsiella*	↓ *S24-7*; ↓ *Lachnospiraceae*; ↓ *Oscillospira*; ↓ *Parabacteroides*	Gu et al., 2022 [30]
Tsumura Suzuki obese diabetes mice feces	Coffee, caffeine and chlorogenic acid	↑ Firmicutes	↓ Bacteroidetes	Nishitsuji et al., 2018 [28]
Mice fecal samples	Coffee and galacto-oligosaccharide	↑ *Bifidobacterium* spp.	↓ *E. coli*; ↓ *Clostridium* spp.	Nakayama & Oishi, 2013 [32]

Notes: spp.—species; ↑: Increased; ↓: Decreased.

### 2.2. Effect of Coffee on Microbiota Diversity

In addition to the microbiota composition, the microbiota diversity level is another important factor accounting for the overall impact of microbiota to its host. In general, higher diversity is associated with good health outcomes, whereas lower diversity is associated with adverse health outcomes, such as a higher risk of colorectal cancer and inflammatory bowel disease (IBD) in the gut [33,34]. A large population-based metagenomics study found that consumption of coffee, tea, and red wine, which all have a high polyphenol content, was associated with increased alpha diversity of gut microbiome in stool samples [35]. A recent cross-sectional study showed that the colonic mucosa-associated bacteria differed significantly in the community composition and structure based on daily caffeine and coffee intake in adults. Dai et al. evaluated the effect of low and high coffee consumption on colonic mucosa-associated microbiota composition using human biopsies and found that higher coffee intake was related to higher alpha diversity and higher relative abundance of *Faecalibacterium* and *Alistipes*, and lower relative abundance of *Erysipelatoclostridium* [36]. It remains to be determined whether caffeine and coffee consumption could affect health outcomes through modulating gut microbiota diversity.

### 2.3. Effect of Coffee on Microbiota Growth

Several studies have also investigated the effects of coffee on the growth of gut microbiota. Jaquet et al. reported that coffee promoted the growth of certain select bacteria species [27]. However, Nakayama et al. found that coffee may exert antimicrobial effect against *E. coli* [32]. There are also reports that fecal bacteria were not affected by coffee consumption [26]. In our recent study on rats, we found that coffee extract significantly suppressed the growth of gut microbiota [31]. Intraluminal fecal contents were collected from the distal ileum and colon of naïve rats. The fecal contents were diluted and cultured on regular and coffee-containing LB agar plates. Compared to regular LB agar, the growth of bacteria from the intraluminal colon content was dramatically suppressed in LB agar with 1.5% coffee (9.31 × 10^9^ CFU/gram vs. 9.65 × 10^9^ CFU/gram). With 3% coffee, the growth of the bacteriome was further suppressed to 5.02 × 10^5^ CFU/gram). A similar inhibitory effect was found for the ileal contents. Decaffeinated coffee extract showed the similar suppressing effect on bacterial growth in vitro. We also determined the in vivo effect of coffee on the total bacteria population. Bacterial culture and quantitative RT-PCR studies found that oral gavage treatment with coffee for 3 days with daily dose of 250 mg significantly suppressed the total number of bacteria in the colon. Again, decaffeinated coffee showed a similar effect to regular coffee. We found that both regular and decaffeinated coffee also reduced Enterobacteria and γ-proteobacteria class in rats dosed with coffee for 3 days, suggesting that coffee components or metabolites other than caffeine are responsible for the observed antibacterial effect [31]. Mannooligosaccharides (MOS) extracted from spent coffee grounds able to exert prebiotic effect on gut microbiota by stimulating the growth of some beneficial genera, such as *Barnesiella*, *Odoribacter*, *Coprococcus*, *Butyricicoccus*, *Intestinimonas*, *Pseudoflavonifractor*, and *Veillonella*, while furfural and polyphenols, which are produced from the spent coffee grounds matrix during hydrolysis, could have an inhibitory effect on other beneficial genera, such as *Faecalibacterium*, *Ruminococcus*, *Blautia*, *Butyricimonas*, *Dialister*, *Collinsella*, and *Anaerostipes* [37]. This suggests that different active components of coffee have a distinct effect on microbial growth, and the method of coffee processing plays a unique role in determining the health benefits or detrimental effects of coffee. In vitro fecal M-batch culture studies also reported that coffee favored the growth of *Bifidobacterium*, *Lactobacilli*, and *F. prausnitzii* in lumen, whereas the XIVa group, *Coprococcus genus* and *F. prausnitzii*, were increased in the mucosal environment, suggesting that coffee has distinct effect on the microbial population, depending upon the microenvironmental factors [38].

## 3. Effect of Coffee on Gastrointestinal Infections and Immunity

The gut microbiota plays a vital role in competitive inhibition of intestine pathogens and helps to sustain intestinal homeostasis and to improve the immune system [39]. As described above, coffee appears to have an indiscriminate antibacterial effect [31]. Moreover, some studies reported that coffee may have the tendency to selectively decrease the abundance of pathogenic bacteria. For instance, coffee consumption significantly decreased the *E. coli* and *Clostridium* spp. population in both the small intestine and the colon of mice [32,40]. Several studies have tried to determine which components in coffee are responsible for its antibacterial effect. Coffee extracts have high contents of chlorogenic acid that have been correlated to the antibacterial, antifungal, and antiviral efficacy of coffee [41]. Phenolic acids and melanoidins, two important metabolites present in coffee, also exerted antimicrobial activity against Gram-positive bacteria (*Staphylococcus aureus*, *Listeria monocytogenes*) and yeast (*Candida albicans*) [42].

Coffee components such as polyphenols can impact both innate and adaptive immune responses in the gut [43,44]. Caffeine suppresses the release of proinflammatory cytokines, which in turn decrease the activity of different immune cells such as natural killer (NK) cells, macrophages, and T and B cell proliferation [44]. Kahweol, another bioactive compound of coffee, mediates anti-inflammatory action by modulating the expressions of macrophage cyclooxygenase-2, nitric oxide synthase, and NF-κB expression [45]. Also, caffeic acid supplementation in mice model of colitis alleviated colonic inflammation by inhibiting proinflammatory cytokines and increasing IL-10 levels, parallelly decreasing the relative abundance of *Bacteroides* and *Turicibacter* and enhancing the relative abundance of *Alistipes* and *Dubosiella* in the gut [46]. In contrast, Ref. [47] reported a positive correlation between coffee consumption and upper GI tract symptoms in patients with active *Helicobacter pylori* infection.

Inflammatory bowel disease (IBD), including Crohn’s disease and ulcerative colitis, is chronic gut inflammation characterized with frequent recurrence. IBD often has complex etiology, and gut microbiota dysbiosis has been implicated in the disease development of IBD. Multiple studies reported that in patients with Crohn’s disease and ulcerative colitis, coffee consumption aggravated symptoms and was associated with the recurrence of IBD [48,49]. On the contrary, others reported that IBD was not associated with coffee. In fact, coffee consumption was reportedly protective on ulcerative colitis [50,51]. Further studies are needed to evaluate whether the coffee effect on IBD is direct or through its impact on microbiota dysbiosis.

Irritable bowel syndrome (IBS) is one of the most common gut disorders in the Western world that has considerable effect on quality of life, healthcare costs, mental health, and social behavior of the affected individual [52]. Altered gut–brain interactions are one of the main characteristics of IBS, with gut dysbiosis proposed as one of the possible causes of IBS [53,54]. Acute bacterial gastroenteritis can cause chronic, low-grade intestinal wall inflammation that is sufficient to alter the gut neuromuscular and epithelial cell function to trigger IBS [55,56]. Caffeine and other bioactive compounds present in coffee can affect multiple pathways that are implicated in IBS pathogenesis. Different studies evaluated the effect of regular coffee consumption on the risk of developing IBS symptoms and reported conflicting results. A recent meta-analysis of available datasets of IBS patients revealed that coffee drinkers had a reduced likelihood of developing IBS compared to controls [57]. Contrastingly, a comprehensive IBS patient survey revealed that increased gut symptoms like diarrhea are associated with caffeine consumption [58]. Further controlled and focused studies are needed to critically evaluate whether caffeine or any other bioactive compounds of coffee have a direct role in developing and exacerbating IBS.

## 4. Effects of Coffee on Gastrointestinal Motility and Secretion

Gastrointestinal motility and secretion are crucial for digestive health, as these functions help mechanical and chemical digestion, and move food and other intraluminal contents at an appropriate pace throughout the alimentary tract. Abnormal motility and secretion may lead to many disorders, including gastroesophageal reflux disease (GERD), peptic ulcer, gastric dyspepsia, and constipation, and functional obstructions such as postoperative ileus.

Coffee is known to stimulate gastric and pancreatic secretion in normal subjects [59,60]. Feldman et al. discovered that decaffeinated coffee stimulated gastric acid secretion, with a response representing 70% of the peak acid output response to pentagastrin. They also found that it increased serum gastrin levels. Decaffeinated coffee was found to be more potent than a then-widely-used protein test meal (Bacto-peptone) in stimulating acid secretion and gastrin release [59]. Acquaviva et al. found that a coffee solution corresponding to 2 cups of homemade coffee significantly increased gastrin level in the blood by 2.3-fold, and decaffeinated coffee had a similar effect with a 1.7-fold increase in gastrin in the blood [61]. Further studies suggest that caffeine and other coffee components may directly act on G-cells to stimulate gastrin release and enhance gastric secretion [62,63]. Coffey et al. confirmed the effect of coffee on gastrin release and found that coffee and decaffeinated coffee stimulated not only gastric secretion but also exocrine pancreatic trypsin secretion [60]

In 1980, Thomas et al. found that coffee significantly decreased lower esophageal sphincter pressure in both normal subjects and patients with reflux esophagitis [64]. Decaffeinated coffee had similar effects to coffee [65,66]. In vitro studies found that coffee stimulated gastric muscle contractions followed by relaxation [67,68]. However, in vivo human studies found that coffee does not significantly alter gastric emptying [69]. Given the effects of coffee on LES pressure and gastric secretion, many studies have looked into the possible links between coffee consumption and risks of gastroesophageal reflux disorder, dyspepsia, and peptic ulcer [49,66]. Current data suggest that low-level coffee consumption (less than 4 cups a day) does not seem to influence the occurrence of GERD [70]. However, higher-level consumption of coffee (more than 4 cups a day) may increase the risk of GERD [70]. There are conflicting data about the links between coffee consumption and risks of functional dyspepsia and peptic ulcer disorders [49].

The effect of coffee on colonic motility is well known [71,72]. A study on 99 healthy volunteers reported that 29% described a compelling need to defecate after the ingestion of a cup of coffee, suggesting colonic stimulation by coffee [71]. Another study showed that the distal colonic motility was increased rapidly within minutes after coffee ingestion [71]. Decaffeinated coffee had a similar effect. These results suggest that coffee may be beneficial in the prevention of constipation. In fact, some population-based studies found that the consumption of coffee is inversely associated with the prevalence of constipation [31]. As gut microbiota may play an important role in modulating gut motility through microbial metabolites such like short chain fatty acids (SCFAs) and tryptophan metabolites, it is possible that coffee-induced alterations in gut microbiota may account for its effect on motility [73]. However, it is undeniable that coffee can directly act on the neuromuscular system to impact gut motility. In the in vitro study of isolated rat colonic smooth muscle strips, we found that regular and decaffeinated coffee dose-dependently (0.1 to 10 mg/mL) stimulated muscle contractions. The coffee-stimulated contractions were not affected by the neural activity blocker tetrodotoxin or nicotinic receptor antagonist hexamethonium, but were completely blocked by muscarinic receptor antagonist atropine. These data suggest that coffee causes muscle contractions directly by acting on muscarinic receptors of the gut smooth muscle cells or inhibiting acetylcholinesterase (an enzyme that breaks down acetylcholine) in a caffeine-independent manner [31]. In the in vivo study, we found that coffee consumption for 3 days led to a moderate increase in ileal smooth muscle contractility and a decrease in Enterobacteria copy numbers. It is not known whether there is any cause–effect relation between the altered gut microbiota and increased ileal motility in the coffee-treated animals [31].

Recent studies found that coffee also significantly impacts small intestinal motility. Postoperative ileus (POI) is a dysmotility condition with significant inflammatory changes that happens after a laparotomy operation. Motility dysfunction and slow transit mainly occur in the small intestine in POI. Multiple studies have reported the benefits of coffee consumption in POI [74]. Different studies have suggested that coffee is a low-cost solution to accelerate postoperative recovery of gut function and motility after colorectal and gynecological surgery [74,75]. Our recent study in a preclinical model found that coffee does not affect inflammatory response in POI in mice, but significantly improves intestinal transit and stimulated ileal smooth muscle contractions in a muscarinic receptor-dependent manner [76]. These effects may contribute to the benefits of coffee in POI.

## 5. Effect of Coffee on the Gut-Microbiota–Brain Axis

The old term gut–brain axis (GBA) refers to the bidirectional communication between the central and the enteric nervous system (CNS and ENS), linking emotional and cognitive centers of the brain with the peripheral gastrointestinal functions [77]. Recent advances in research have identified the importance of gut microbiota in influencing the gut–brain axis by bidirectional signaling between the gut microbiota and brain through neural, endocrine, and immunological processes, a new term “gut-microbiota–brain axis” emerges [78]. It refers to the network of connections that passes bidirectional communication between gut microbiota and the brain, and is crucial in maintaining homeostasis of the gastrointestinal, central nervous, and microbial systems.

It is possible that coffee-induced alterations in gut microbiota composition may translate into changes in effector molecules involved in the GBA signaling, affecting multiple neurological and psychological processes. A randomized, double-blind, crossover clinical trial with 40 healthy people in nonstressful conditions reported that acute coffee consumption was not associated with any gastrointestinal symptoms but activated the sympathetic nervous system, associated with increases in salivary α-amylase, but not salivary cortisol. This was interpreted as due to a possible antistress effect of coffee mediated by metabolites of coffee other than caffeine [79]. Another study demonstrated that severe sleep loss caused the accumulation of reactive oxygen species (ROS) in the gut but not in the brain, and this effect could be prevented by the administration of an antioxidant such as melanoidins from coffee [80]. Recent studies tried to correlate the coffee-induced gut dysbiosis to different brain functions. Caffeine-induced sleep restriction in mice was associated with altered diversity and composition of the intestinal microbiota, i.e., reduced Proteobacteria and Actinobacteria abundance, and altered the metabolic profiles of the gut microbiome [81]. A recent study in chronic paradoxical sleep deprivation (PSD) rats revealed that both coffee and decaffeinated coffee significantly improved the depression-like behaviors with increased levels of superoxide dismutase and glutathione peroxidase and decreased levels of IL-6 and TNF-α in the serum. Parallel gut microbiota analysis showed that coffee intake reversed the gut dysbiosis and restored the abundance of *S24-7*, *Lachnospiraceae*, *Oscillospira*, *Parabacteroides*, *Akkermansia*, and *Klebsiella* to a healthy level, suggesting a close relationship among coffee, gut microbiota, and CNS [30].

A study conducted on 62 healthy people showed that routine dietary caffeine intake increased pain threshold, heat pain tolerance, and pressure pain threshold through a mechanism involving peripheral adenosine receptors and cyclooxygenase pathway [82]. Gut microbiota has been implicated in altering both pathways, suggesting a close interplay between microbiota, caffeine, and pain perception [83,84]. However, further research is needed to understand the molecular crosstalk between gut microbiota, ENS, and CNS.

## 6. Effect of Coffee on Absorption and Nutrition

The gut microbiota has a pivotal role in the metabolism and absorption of different minerals and micronutrients. Hence, any change in the gut microbiota due to coffee consumption may indirectly affect the nutrient absorption (Table 2 and Table 3). However, the research outcomes on the role of coffee in nutrient absorption are quite divided. There are studies showing the beneficial effect of moderate coffee consumption on nutrient absorption. However, consuming a large amount of coffee (>4 cups per day) was found to be interfering with nutrient and mineral absorption. In a pathological model of obesity where rats were fed with a high-fat diet, moderate coffee consumption for 10 weeks decreased body weight, adiposity, liver triglycerides, and energy intake. In addition, it also reversed the increase in high-fat associated *Clostridium* Cluster XI groups and increased the levels of Enterobacteria [85]. Caffeine intervention also improved the serum lipid disorders and insulin resistance in a mouse model of metabolic syndrome [86]. These changes were attributed to the change in relative abundance of *Dubosiella*, *Bifidobacterium*, and *Desulfovibrio* and decreased *Bacteroides*, *Lactobacillus*, and *Lactococcus*. Moreover, caffeine supplementation in high-fat-diet mice also altered serum metabolomics by altering lipid metabolism, bile acid metabolism, and energy metabolism [86]. The coffee-mediated alleviation of metabolic syndrome was also supported in another study that looked at coffee intake in mice [28]. A decrease in *Prevotella* was observed in the coffee-treated group. It is interesting to note that *Prevotella* is normally found in obese humans and implicated in the degradation of dietary fibers. The decrease in *Prevotella* led to an improvement in endotoxemia and systemic inflammation in diabetic mice [28]. This same study found an increase in *Coprococcus* and *Blautia* with coffee treatment. *Corpococcus* parallels fasting levels of gastrointestinal polypeptides in obese women. These peptides are known to stimulate insulin secretion, and this may help to protect against metabolic syndrome. The higher levels of *Blautia*, though, were seen in rats fed with a high-fat diet and are reported in obese individuals. However, *Blautia* has also been found to increase with the consumption of whole grains, contributing to an anti-inflammatory effect, and has been reported to be involved in acetate production [87]. Coffee-mediated changes in gut microbiota are also associated with a reduction in obesity and an improvement in glucose tolerance [88].

The intake of coffee was found to increase the absorption of aspirin by modifying the gut microbiome α- and β-diversities [95]. The increased aspirin absorption was in alignment with decreased abundance of Proteobacteria, Helicobacteriaceae, and Bacteroidaceae populations in the fecal microbiota composition, and with increased Lactobacillaceae population [95].

Coffee consumption in healthy adolescents was found to increase HDL-cholesterol and vitamin D levels [89]. In contrary, coffee intake may be associated with decreased levels of vitamin B concentrations in healthy individuals [91,93]. In animal studies, coffee intake reduced levels of zinc and calcium [90,91]. It is found that drinking a cup of coffee or other caffeinated beverage with an iron-rich meal may decrease iron absorption by 39–90% [96,97]. Coffee intake also seems to be associated with decreased levels of vitamin B concentrations in healthy individuals [91,93]. Future studies should investigate the effect of consumption time of coffee in relation to the intake of these nutrients, as some studies show that iron consumption is not decreased when coffee is consumed an hour before a meal [92]. Coffee may also decrease the absorption of glucose in the intestines [96]. For the general population, this may have little impact upon moderate coffee consumption. However, for individuals who undergo diabetes management or have insulin sensitivity, this decrease in glucose absorption can be beneficial in modulating blood sugar levels. It is not clear whether the effects of coffee on the absorption of vitamins and minerals are mediated by coffee-associated microbiota changes in the gut, as it has not been thoroughly investigated.

## 7. Coffee and Medication Interaction

While changes in gut microbiota from coffee may impact the absorption of different nutrients and substances that enter the body, these findings also guide us towards coffee’s potential effects on drug interactions and metabolism (Table 4). Several studies shine light on how coffee, taken either before, with, or after medications, can alter therapeutic effects. The intake of coffee was found to increase the absorption of aspirin by modifying the gut microbiome α- and β-diversities. The increased aspirin absorption was in alignment with decreased abundance of Proteobacteria, Helicobacteraceae, and Bacteroidaceae populations in the fecal microbiota composition, and with increased Lactobacillaceae population [95]. There are many identified minerals and polyphenols in coffee, like caffeine, cholinergic acid, and trigonelline, that result from the breakdown of coffee by the microbiome and can have powerful effects [98]. One of caffeine’s benefits, for instance, was observed in an amoxicillin-dysbiosis study; it was found that caffeine had the potential to slow the growth of Burkholderiaceae in vitro in caffeinated coffee-supplemented mice after antibiotic treatment [29]. Trigonelline, an alkaloid, can inhibit *Citrobacter freundii*, preventing choline breakdown, and, thus, the production of a proatherosclerotic metabolite responsible for cardiovascular risk [99]. Even cholinergic acid in conjunction with a Chinese medicine, Geniposide, was found to be therapeutic for nonalcoholic fatty liver disease (NAFLD) in mice by protecting gut barrier function along with other mechanisms [100].

Gut bacteria can also metabolize certain compounds into metabolites that may either induce or inhibit CYP enzymes. A study conducted on healthy male human volunteers showed that coffee administration resulted in paracetamol being largely cleared from the blood by hepatic cytochrome P-450 and P-448 [96]. Because of its enzyme-inducing ability, caffeine can lower the concentration of the drug in the plasma in certain conditions. The most abundant enzyme that is responsible for metabolizing caffeine is a CYP450 liver enzyme, which is also an enzyme that plays a major role in the clearance of many medications. A greater amount of coffee, however, can saturate these enzymes and increase serum concentration levels of the drugs if taken at the same time. A pharmacokinetic study performed on healthy volunteers showed that the plasma concentration of clozapine (antischizophrenic medication) was increased by 97% after concomitant administration of 2–3 cups of coffee; the blood concentrations of lithium, theophylline, warfarin, and several antidepressants and antipsychotics drugs also increased and were followed by variable consequences, more commonly liver toxicity [102].

These drug metabolizing enzymes can oftentimes be stimulated by short chain fatty acids (SCFAs), like acetate, propionate, and butyrate, produced from dietary fibers fermented by the gut microbiota after coffee intake. SCFAs can have direct effects by inducing other enzymes or alter the expression of drug transporters in the liver and intestines, affecting the absorption and metabolism of certain drugs. Togao et al. found that a higher abundance of bacteria, such as *Clostridium butyricum* producing high levels of butyrate, may enhance CYP3A activity [103]. Because CYP3A metabolizes more than 50% of marketed drugs, it may respond sensitively to gut-microbiome-targeted interventions if extra butyrate or other SCFAs are produced from coffee consumption. This may affect inflammatory bowel disease (IBD) patients, as butyrate uptake and utilization by colonocytes is impaired in IBD patients. SCFAs can also directly induce therapeutic effects, bridging the role of the enteric nervous system with the rest of the body. Poll et al. found that acetate affects blood pressure and cardiac function through parallel mechanisms and establishes a role for SCFAs in modulating sympathetic function and the contractility of the heart [104].

## 8. Effect of Coffee on Oral Microbiome

A healthy adult human mouth contains a complex and resilient ecosystem of hundreds of different microbial species that are collectively referred to as the salivary microbiome (or oral microbiome). The salivary microbiome is considered to be very similar to the gut microbiome in terms of complexity and diversity, and it is implicated in oral health and functional and infectious diseases [105]. Coffee consumption has been shown to have a significant effect on oral microbiome composition. Coffee inhibited the overall growth of salivary bacteria when cultured in vitro, along with a reduction in *Streptococcus salivarius* and a proportional increase in *Streptococcus mitis* and *Streptococcus infantis* [106]. A large human volunteer study found that high coffee intake was associated with greater abundance of *Granulicatella* and *Synergistetes* in the oral cavity [107]. Murugesan et al. evaluated the effect of coffee consumption in healthy adults in detail [108]. It was found that Actinobacteria, Firmicutes, Proteobacteria, and Saccharibacteria phyla had significantly higher abundance in the saliva samples from coffee drinkers compared to the noncoffee drinkers. The salivary microbiome of coffee drinkers was also significantly more diverse with higher alpha diversity compared to noncoffee drinkers. Further, a small population study containing 43 Korean subjects also found that coffee drinking significantly improved alpha diversity and significantly altered beta diversity of the oral microbiome compared to noncoffee drinkers. The study also found that *Oribacterium*, *Campylobacter*, and *Megasphaera* were abundant in the oral microbiome of coffee consumers [109]. It has been reported that low to moderate coffee consumption can be beneficial to people with periodontitis, which is induced by an imbalance between the oral microbiota and the immune system, and coffee enhances the richness of the oral microbiome, aiding in restoring the balance between the microbiome and the immune system [110]. However, high amounts of coffee consumption were positively associated with periodontal disease etiology/progression [111]. In addition, it has been observed that oral microbiome richness significantly decreased in individuals consuming high amount of coffee everyday (>5 cups) [112]. Further studies are needed to evaluate the benefits and risks of coffee consumption and specific components of coffee on different oral diseases and infections.

## 9. Summary and Conclusions

Growing evidence suggests that coffee consumption alters gut microbiota composition and growth. Moderate consumption of coffee (<4 cups per day) is associated with higher microbiota diversity, increased relative abundance of beneficial bacteria phyla such as Firmicutes and Actinobacteria, and decreased Bacteroidetes. Coffee may also have antibacterial activities in the gut, and phenolic acids and chlorogenic acid may be partly responsible for the effect. Caffeine has anti-inflammatory properties, and some other coffee components such as phenolic acid may have impacts on immune responses.

Coffee and decaffeinated coffee stimulate gastrin release and gastric and pancreatic secretion. They are also found to decrease the lower esophageal pressure. High consumption of coffee (>5 cups per day) may increase the risk of gastro-esophageal reflux disease. Coffee is found to increase ileal and colonic motility in a caffeine-independent manner. The stimulating effect is partly through the action of coffee on muscarinic receptors in gut smooth muscle cells. Coffee may also act on the gut–brain axis to impact visceral pain sensation and even CNS functions. There is some evidence that these effects may be associated with the altered gut microbiota.

Coffee intake is found to alter lipid absorption, possibly through modification in the gut microbiota. Coffee consumption may also increase the levels of HDL-cholesterol and vitamin D but reduce the levels of zinc, calcium, and vitamin B. Coffee consumption may also affect drug interaction and metabolism. It has been found that coffee increases the absorption of aspirin by modifying the gut microbiome α- and β-diversities.

Studies have shown that coffee consumption has significant effects on oral microbiome composition and diversity. Moderate coffee consumption is associated with higher diversity of the oral microbiota community. However, a high amount of coffee intake has been implicated in the progression of periodontal diseases.

Taken together, current studies have presented many conflicting results regarding the impacts of coffee in the gastrointestinal tract. Further research on the effects of coffee on gut microbiota and bowel functions is warranted.

## Figures and Tables

**Table 2:** Coffee-associated changes of metabolic components and absorption via microbiota change.

Design	Treatment	Significance	Microbiota Change	Reference
In vivo rats	Coffee	Reduction in liver triglycerides	*Clostridium* Cluster XI ↓	Cowan et al. 2014 [85]
In vivo rats	Coffee	Reduction in obesity, metabolic syndrome, and inflammation; increase in gut barrier function	Firmicutes (F)-to-Bacteroidetes (B) ratio ↓	Cowan et al. 2014 [85]
In vivo rats	Coffee	Potential for gut dysbiosis, antibiotic resistance, opportunistic infection; may be involved with insulin resistance	Enterobacteriaceae ↑	Cowan et al. 2014 [85]
In vivo mice	Caffeine	Protection of gut lining, barrier function, and production of SCFAs	*Dubosiella*, *Bifidobacterium* and *Desulfovibrio* ↑	Chen et al. 2023 [86]
In vivo mice	Caffeine	Reduction in nutrient breakdown and immune modulation, but potentially, a restoration from dysbiosis	*Bacteroides*, *Lactobacillus* and *Lactococcus* ↓	Chen et al. 2023 [86]
In vivo mice	Coffee	Improvement in endotoxemia and systemic inflammation	*Prevotella* ↓	Nishitsuji, Watanabe, & Xiao 2018 [28]
In vivo mice	Coffee	Increase in gastrointestinal polypeptide; stimulation of insulin secretion and protection against metabolic syndrome	*Coprococcus* ↑	Nishitsuji, Watanabe, & Xiao 2018 [28]
In vivo humans In vivo mice	Coffee	Support in acetate production, but a marker of high-fat diet	*Blautia* ↓	Martinez et al. 2013 [87]; Nishitsuji, Watanabe, & Xiao 2018 [28]

Notes: ↑: Increased; ↓: Decreased.

**Table 3:** Effects of coffee on the intestinal absorption of nutrients.

Design	Treatment	Nutrient Effects	Mechanism	References
In vivo human	Coffee	Circulating Vitamin D ↑	Inhibition of Vitamin D receptors	Al-Othman et al. 2012 [89]
In vivo human	Caffeine	Calcium levels ↓	Imbalance of Calcium/inhibition of Vitamin D receptors	Barger-Lux & Heaney 1995 [90]
In vivo human	Coffee	Zinc levels ↓	Binding of phenolic compounds	Pécoud et al. 1975 [91]
In vivo human	Coffee	Iron absorption ↓	Phenolic binding of nonheme iron in the lumen	Morck 1983 [92]
In vivo human	Coffee	B vitamin levels ↓	Complex formation with polyphenols	Ulvik 2008 [93]
In vivo human	Coffee	Glucose ↓	Inhibition of hepatic glucose-6-phosphate translocase	Ohnaka 2012 [94]

Notes: ↑: Increased; ↓: Decreased.

**Table 4:** Summary of coffee consumption and drug interactions.

Design	Treatment	Medication Interaction	Mechanism	References
In vivo mice	Coffee	Aspirin absorption ↑	Proteobacteria, Helicobacteraceae, and Bacteroidaceae ↓; Lactobacillaceae ↑	Kim et al. 2022 [95]
In vivo human	Coffee	Paracetamol Absorption ↑	Competitive binding to adenosine receptors	Iqbal 1995 [101]
In vivo human	Coffee	Clozapine, Lithium, Theophylline, Warfarin Absorption ↓	Activation of CYP enzymes by metabolites *	Belayneh et al. 2020 [96]
In vitro mice	Coffee	Amoxicillin	Slowed growth of Burkholderiaceae	Diamond et al. 2021 [29]
In vivo mice	Cholinergic acid	Geniposide absorption ↑	Protect gut barrier function	Peng et al. 2018 [100]

Notes: * Gut microbiota, such as *Clostridium butyricum*, that promote butyrate production can enhance CYP3A activity. ↑: Increased. ↓: Decreased.
